# Supplementary material for: QUIRKY regulates root epidermal cell patterning through stabilizing SCRAMBLED to control CAPRICE movement in Arabidopsis
Source: Nat Commun. 2019 Apr 15;10:1744. doi: 10.1038/s41467-019-09715-8 (PMC6465271; doi:10.1038/s41467-019-09715-8)
Supplement: Supplementary file 1 — Supplementary Information [file 41467_2019_9715_MOESM1_ESM.pdf]

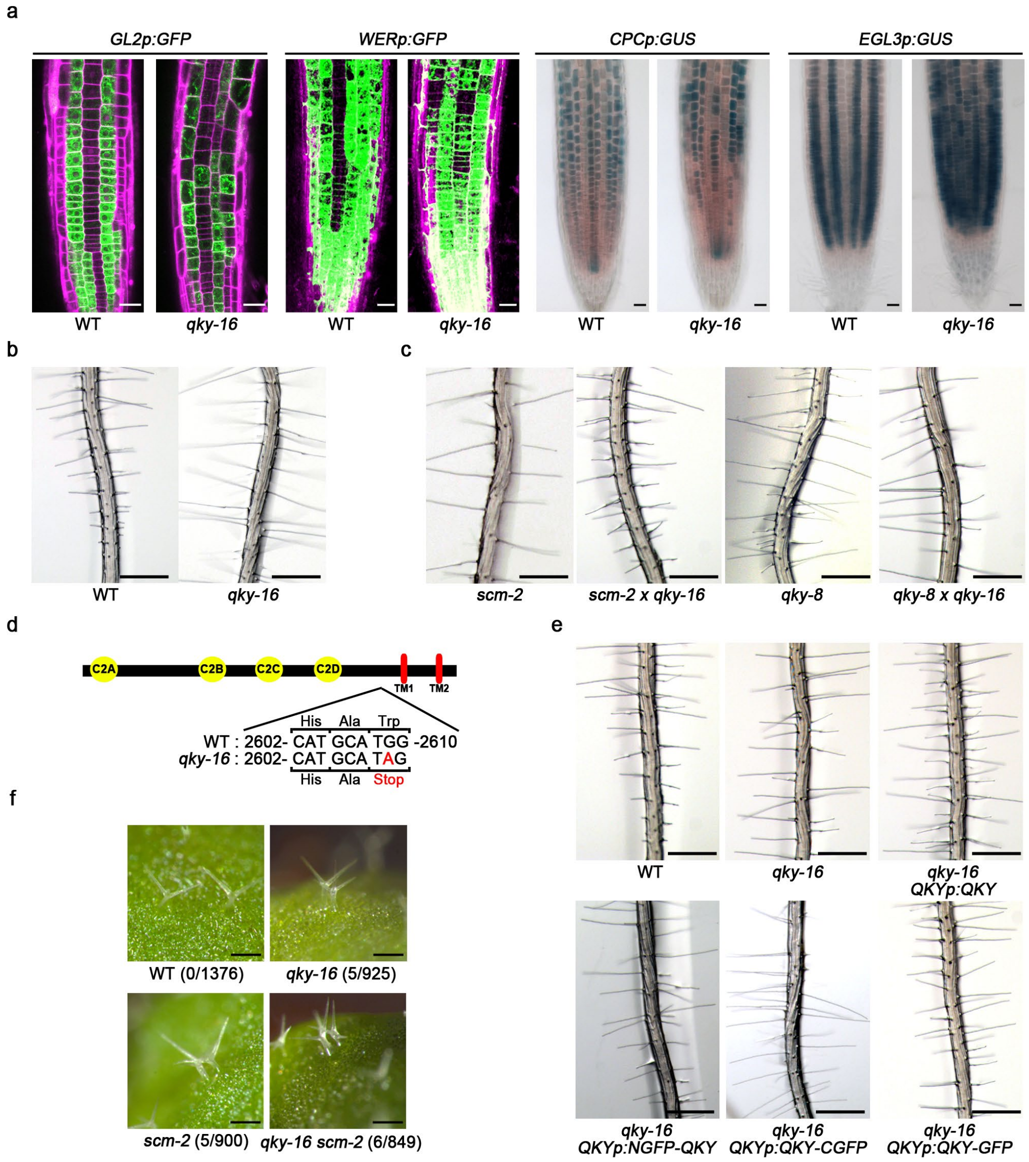

Supplementary Figure 1. A new allele of *qky*.

a, The promoter activity of *GL2* (*GL2p:GUS*), *WER* (*WERp:GFP*), *CPC* (*CPCp:GUS*) and *EGL3* (*EGL3p:GUS*) in the root epidermis of the *qky-16* mutant. Bar = 25 µm. **b,** Four-daly-old root phenotypes of the *qky-16* mutant. Bar = 250 µm. **c,** Allelism test with *scm-2* and *qky-8*. Four-day-old root phenotype of *scm* and *qky* mutants, and of F1 progenies from the cross between them. Bar = 250 µm. **d,** Predicted domain structure of the QKY protein. Four C2 domains and the non-sense mutation site in the *qky-16* allele are shown. Numbers indicate the nucleotide sequence position in the open reading frame. **e,** Complementation analysis of the *qky-16* mutant using a *QKY* genomic DNA fragment (*QKYp:QKY*) and the *QKY* translational fusion constructs. GFP was fused to the N-terminus or C-terminus of QKY, or

inserted between the first transmembrane domain and the phosphoribosyltransferase domain, and expressed under the control of the QKY regulatory sequences (*QKYp:NGFP-QKY*, *QKYp:QKY-CGFP*, and *QKYp:QKY-GFP*, respectively). Bar = 250 μm. **f,** Twin trichomes in the wild type and mutants. Trichomes on the adaxial surface of fully expanded first and second leaves were counted. Numbers in parenthesis indicate the number of twin trichomes / the number of total trichomes observed. Statistical significance of differences in the frequency was shown by chi-square test, in which the actual *P* values for the differences between the wild type and the *qky-16* mutant, between the wild type and the *scm-2* mutant, and between wild type and the *qky-16 scm-2* double mutant are 0.0063, 0.0056 and 0.0018, respectively. However, there were no statistically significant differences between the double mutant and either the *qky-16* or the *scm-2* mutant (*P* = 0.6560 and 0.6894, respectively).

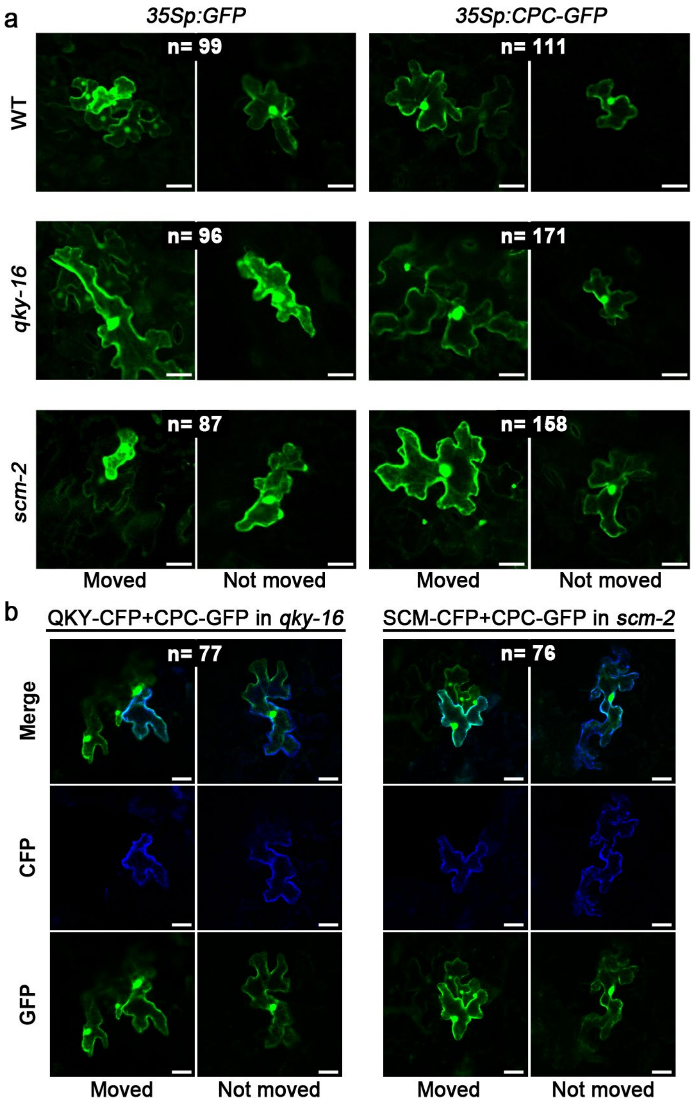

Supplementary Figure 2. Intercellular movement of CPC-GFP in the leaf epidermis. Confocal images of the epidermal cells of Arabidopsis rosette leaves transiently expressing *CPC-GFP*, *SCM-CFP*, and *QKY-CFP*. **a,** *GFP* or *CPC-GFP* are expressed for 4h after introducing *35Sp:GFP* or *35Sp:CPC-GFP* by particle bombardment. The total number of observed cells are indicated in each panel. **b,** *35Sp:CPC-GFP* is co-bombarded with *35Sp:SCM-CFP* or *35Sp:QKY-CFP* into rosette leaves of the indicated mutants by particle bombardment. Their co-expression and the movement of CPC-GFP were analyzed after 4 h of incubation. GFP fluorescence appears in green and CFP fluorescence in cyan. Bar = 50 μm.

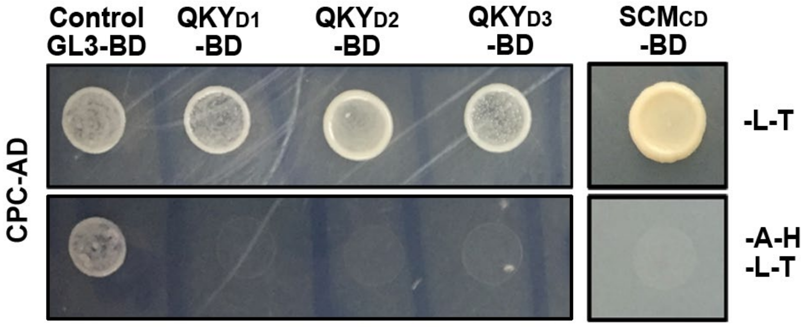

Supplementary Figure 3. Interaction test between CPC and QKY, and between CPC and SCM.

Interactions of CPC with various domains of QKY, and the SCM cytoplasmic domain were analyzed with the yeast two-hybrid assay. The indicated combinations of constructs were co-introduced into the yeast strain AH109. Transformants were grown on -L-T (lacking leucine and tryptophan) control plates and -A-H-L-T (lacking adenosine, histidine, leucine, and tryptophan) selective plates for 3 days. The combination of CPC/GL3 was used as positive control.

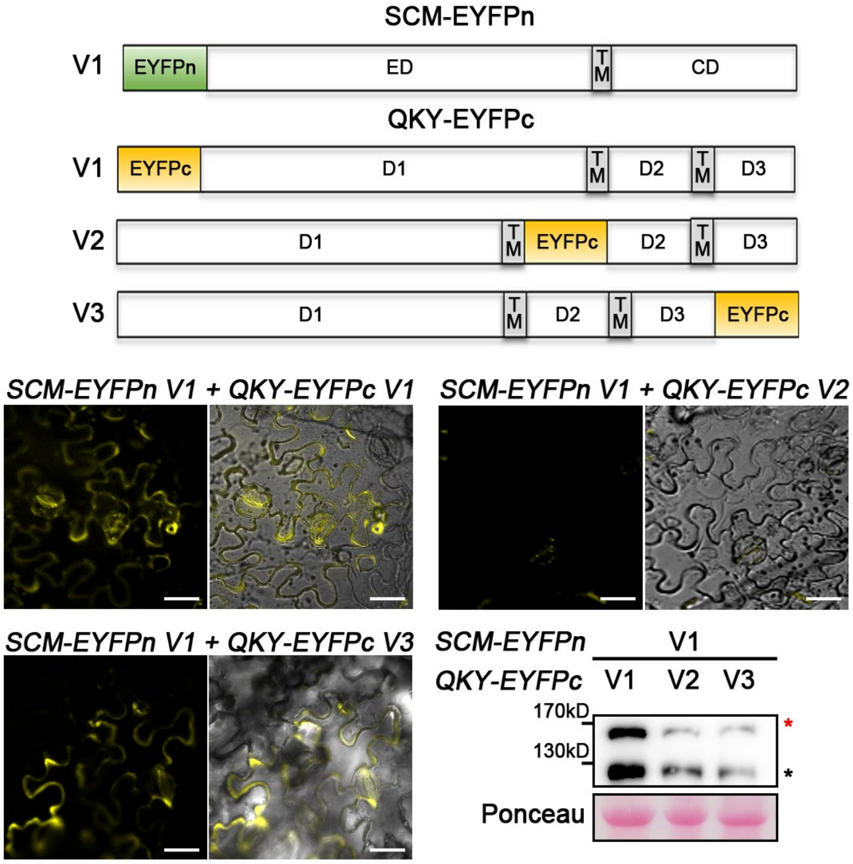

Supplementary Figure 4. Bimolecular fluorescence complementation assay with QKY and SCM proteins.

SCM-EYFPn-V1 in which the N-terminal half of EYFP (EYFPn) was fused to the N-terminus of SCM which is known as an extracellular domain and QKY-EYFPc-Vx in which the C-terminal half of EYFP (EYFPc) was fused to various sites as shown in the upper panel were co-expressed in the *Nicotiana benthamiana* leaf epidermal cell using a biolistic particle delivery system. Their expression was confirmed by Western blot analysis with total protein extracts using polyclonal anti-GFP antibodies. The red and the black asterisk indicate the QKY-EYFPc fusion protein and the SCM-EYFPn fusion protein, respectively. Bar = 25 μm.

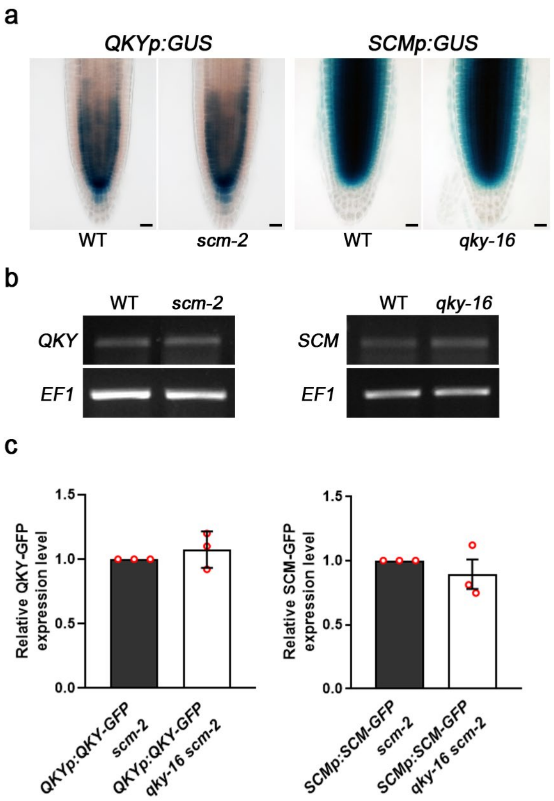

Supplementary Figure 5. Transcriptional regulation between *QKY* and *SCM*.

a, *QKYp:GUS* and *SCMp:GUS* reporter expression in each mutant background. Histochemical GUS staining of *QKYp:GUS* and *SCMp:GUS* shows their expression in the root meristem. Bar = 25 µm. **b,** Relative levels of the *QKY* transcripts (left) and the *SCM* transcripts (right) in wild-type and mutant roots were examined by semi-quantitative reverse transcription (RT) -PCR. Total RNA was extracted from 4-day-old seedlings using the RNeasy kit (Qiagen), and used for the RT-PCR analysis. The *EF1* gene was used as internal control. The primers used for this RT-PCR analysis are listed in Supplementary Table 1. **c.** Quantitative real-time RT-PCR analysis showing the levels of the QKY-GFP (left) and the SCM-GFP transcripts (right). Average values of the relative expression levels from 3 independent experiments are shown in a graph and the error bars indicate standard deviations. Quantitative real-time RT-PCR with the total RNAs extracted from the root tips of the *SCMp:SCM-GFP scm-2* seedlings and the *SCMp:SCM-GFP qky-16 scm-2* seedlings did not show any significant difference in the SCM-GFP transcript level. The steady-state level of QKY-GFP transcripts was similar between in the *QKYp:QKY-GFP scm-2* root and in the *QKYp:QKY-GFP qky-16 scm-2* root.

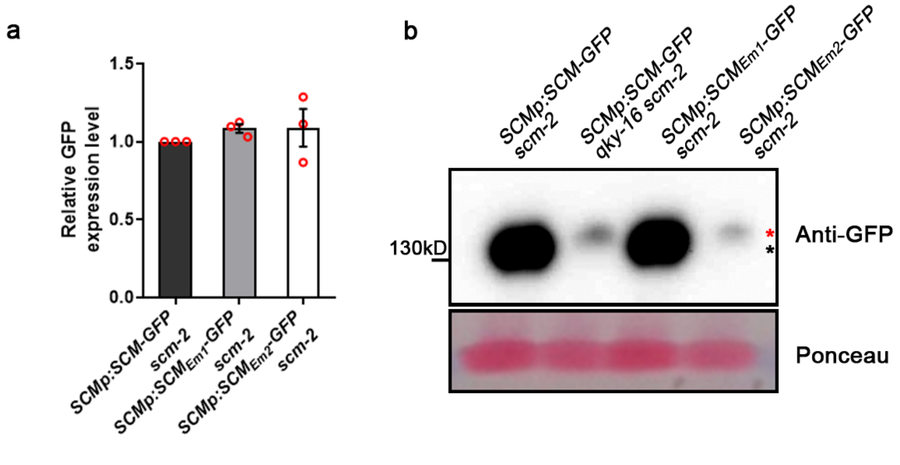

Supplementary Figure 6. Effect of mutations in the SCM PRD on the steady-state level of its transcript and protein.

a, Quantitative real-time RT-PCR analysis with the total RNAs extracted from the root tips of the *SCMp:SCM-GFP scm-2* seedlings, the *SCMp:SCM*$_{Em1}$*-GFP scm-2* seedlings and the *SCMp:SCM-GFP*$_{Em2}$ *scm-2* seedlings did not show any significant difference in the SCM-GFP transcript level between these three plant lines. Average values of the relative expression levels from 3 independent experiments are shown in a graph and the error bars indicate standard deviations. **b,** Western blot analysis using anti-GFP antibodies to examine the SCM-GFP level in the roots of the *SCMp:SCM-GFP scm-2* seedlings, the *SCMp:SCM-GFP qky-16 scm-2* seedlings, the *SCMp:SCM*$_{Em1}$*-GFP scm-2* seedlings and the *SCMp:SCM-GFP*$_{Em2}$ *scm-2* seedlings. The SCM-GFP protein level in the roots of the *SCMp:SCM-GFP*$_{Em2}$ *scm-2* seedlings is much lower than the levels in the roots of the *SCMp:SCM-GFP scm-2* seedlings and the *SCMp:SCM*$_{Em1}$*-GFP scm-2* seedlings. Note that the protein level in the roots of the *SCMp:SCM-GFP*$_{Em2}$ *scm-2* seedlings is similar to the level in the roots of the *SCMp:SCM-GFP qky-16 scm-2* seedlings. Furthermore, the band (SCM$_{Em2}$-GFP) reacted with anti-GFP antibodies has the higher MW (red asterisk) than the SCM-GFP and the SCM$_{Em1}$-GFP (black asterisk), which is the similar to the size of the ubiquitinated SCM-GFP detected in the *qky-16* mutant.

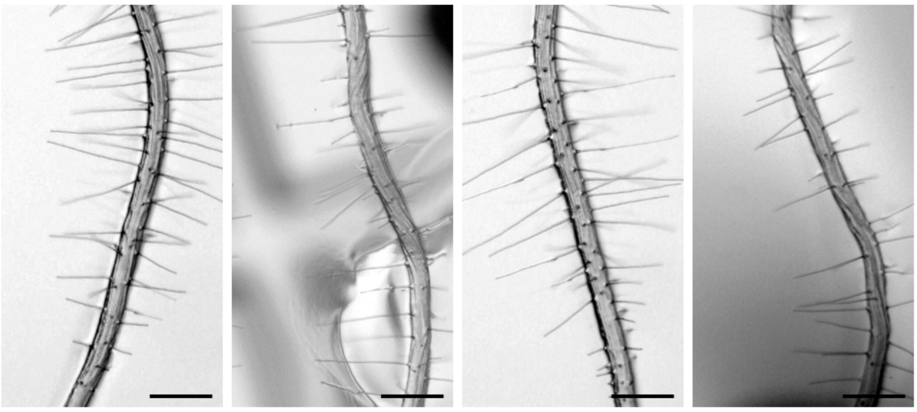

Supplementary Figure 7. Complementation analysis of the *scm-2* mutant phenotype with a mutant version of *SCM*. Images show the root hair phenotype of *scm-2* seedlings harboring different complementation constructs. A *SCM* construct with all 22 lysine codons in the cytoplasmic domain replaced with arginine codons (*SCMp:SCM*$_{m22KR}$*-GFP*) is not able to rescue the abnormal root phenotype of the *scm-2* mutant. Bar = 250 μm.

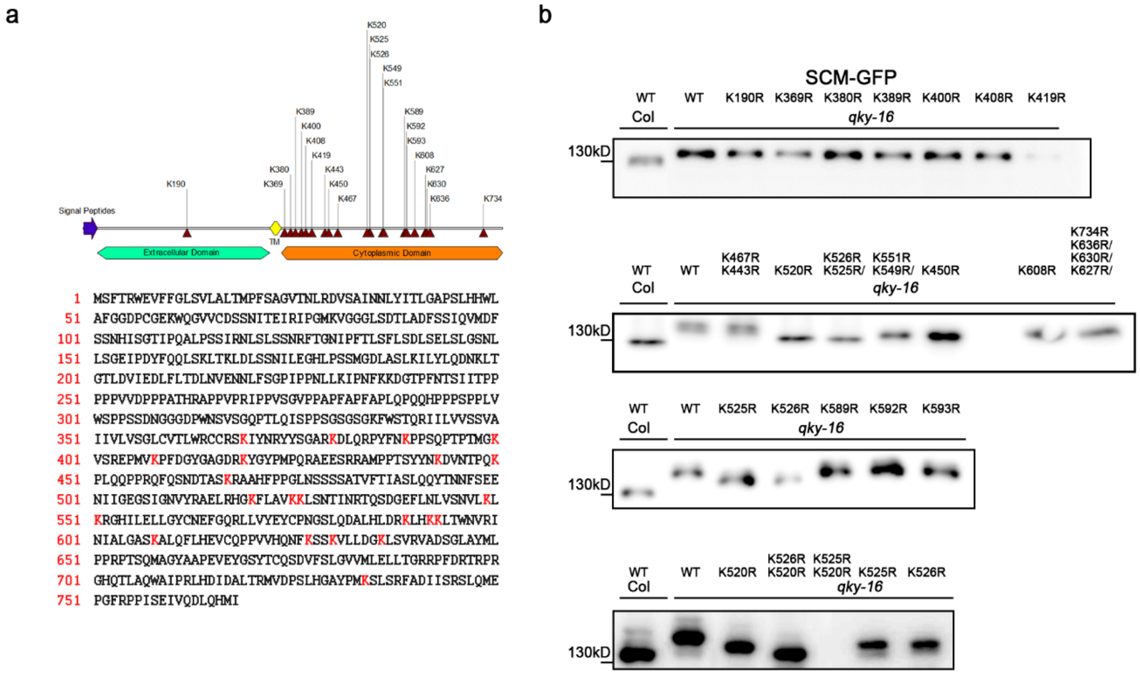

Supplementary Figure 8. Identification of ubiquitination sites in SCM protein.

a, Lysine residues in the cytoplasmic domain of SCM. Twenty-two lysine residues marked red in the cytoplasmic domain of SCM or the lysine residue at 190th in the extracellular domain were replaced by arginine individually, or as a small group. **b,** Western blot analysis of total protein extracts from Arabidopsis rosette leaves transiently expressing the wild-type (WT) or mutated versions of SCM using particle bombardment. The numbers in each lane indicate the replaced residue sites.

Supplementary Table 1. Primers used in this study.

Primer		Sequence
**For reporter gene construct**		
RHD3 promoter	forward	CTGCAGTTCACAACAACCAACCCGTG
	reverse	ACTAGTATCTGCAACGTCCAAGCAATAA
QKY coding region	forward	ACTAGTATGAACACGACGCCGTTTCA
	reverse	TCTAGATTAGATGAGTCGATCGGACA
QKY promoter	forward	CTGCAGGAAAGCAATGTTGTCTCTCT
	reverse	CCCGGGCGTAGTGGTAGTCTTCTCAT
GFP coding region	forward	ACTAGTATGAGTAAAGGAGAAGAACT
	reverse	TCTAGATTATTTGTATAGTTCATCCA
QKY terminator	forward	TCTAGAGGAGAGAAAGATGTCAAGATAG
	reverse	TCTAGAGTTTCTTCCACTTGGCATCA
GUS coding region	forward	ACTAGTATGGTCCGTCCTGTAGAAAC
	reverse	TCTAGATCATTGTTTGCCTCCCTGCT
QKY-N-terminus	forward	ACTAGTATGAACACGACGCCGTTTCA
	reverse	TCCTCCTCCTCCTCCTCCTCCTCCGTAGTACCAAACTCCGATCA
QKY-C-terminus	forward	GCATGGATGAACTATACAAACGGTTTAGACCCAAGATACC
	reverse	TCTAGATTAGATGAGTCGATCGGACA
mGFP5 coding region	forward	GGAGGAGGAGGAGGAGGAGGAGGAATGAGTAAAGGAGAAGAACT
	reverse	TTTGTATAGTTCATCCATGC
SCM promoter	forward	GCGGCCGCTCTAGAACTAGTCTCGGAGTCAACAGAGTACA
	reverse	CTTCCCATCTTGTAAAGCTCATAACTTCAGCCACTGAAGATG
SCM-GFP coding region	forward	ATGAGCTTTACAAGATGGGAA
	reverse	TTAGTCCAAGGTTGTGTATCTTATTTGTATAGTTCATCCA
SCM terminator	forward	GATACACAACCTTGGACTAAG
	reverse	AGATCGGGAATTCCTGCAGCTACTATTTGCGTAAAGAAGAAG
**For tissue-specific QKY expression**		
CO2 promoter	forward	CCCCTGCAGTAACTCCATTATTTACGACT
	reverse	CCCACTAGTAAACTCTTGTTGCATTATTG
SCR promoter	forward	CCCCTGCAGCAATTTTGAATCCATTCTCA
	reverse	CCCCCCGGGGGAGATTGAAGGGTTGTTGG
SHR promoter	forward	CCCCTGCAGGCAACACTCGATAGGTTTCG
	reverse	CCCACTAGTTTTTAATGAATAAGAAAATG
WER promoter	forward	CCCCTGCAGCAAAAACGAATAATTAAAAT
	reverse	CCCACTAGTTCTTTTTGTTTCTTTGAATG
**For RT-PCR analysis**		
QKY	forward	ACCAGCAACAATTTCATCCTCCGC
	reverse	TTGGACCTTGCATTACATGCTGCG
SCM	forward	GTTGCAGACAGCGGTTTGGCTTAT
	reverse	TGCGTCCAGTGAGCAGTTCTAACA
EF1	forward	TGAGCACGCTCTTCTTGCTTTCA
	reverse	GGTGGTGGCATCCATCTTGTTACA
GFP	forward	ATCAAAGCCAACTTCAAGACCCGC
	reverse	AGGGCAGATTGTGTGGACAGGTAA
**For Yeast two-hybrid experiment**		
SCMED-AD	forward	GGATCCGGATGAGCTTTACAAGATGGGA
	reverse	CTCGAGTTATCTTTGAGTGGACCAGAATT
SCMCD-AD	forward	GGATCCGGTGGAGATGTTGCAGAAGTAA
	reverse	CTCGAGTTAGATCATATGTTGAAGATCTT
SCMEm1-AD	forward	ACATCGATTATAACAGGAGGAGGAGGAGGA
	reverse	TCCTCCTCCTCCTCCTGTTATAATCGATGT
SCMEm2-AD	forward	TGTGGTTGATGGTGGTGGCGCTACTCACCG
	reverse	CGGTGAGTAGCGCCACCACCATCAACCACA
QKYD1-BD	forward	CCATGGACATGAACACGACGCCGTTTCA

	reverse	CCCGGGTTAAAGAACCAGATATAGAATAT
QKYD2-BD	forward	GGATCCGGTGGAGATGTTGCAGAAGTAA
	reverse	CTCGAGTTAGATCATATGTTGAAGATCTT
QKYD3-BD	forward	CCATGGCGGCTCTAGGGTTTTATTATCT
	reverse	CCCGGGTTAGATGAGTCGATCGGACAAGC
CPC-AD	forward	GAATTCGGATGTTTCGTTCAGACAAGG
	reverse	CTCGAGTCATTTCCTAAAAAAGTCTCTTCG
CPC-BD	forward	GAATTCATGTTTCGTTCAGACAAGG
	reverse	CTGCAGTCATTTCCTAAAAAAGTCTCTTCG
**For BiFC experiment**		
QKY-V1	forward	AACACGACGCCGTTTCACT
	reverse	CTTAGAATTCCCGGGTTAGATGAGTCGATCGGACAAG
QKY-fragment_1-V2	forward	GGCTGCAGGTCGACGATGAACACGACGCCGTTTCACT
	reverse	GCCTCCACCACCTCCCCGGTAGTACCAAACT
QKY-fragment_2-V2	forward	TTTAGACCCAAGATACCGGC
	reverse	CTTAGAATTCCCGGGTTAGATGAGTCGATCGGACAAG
QKY-V3	forward	GGCTGCAGGTCGACGATGAACACGACGCCGTTTCACT
	reverse	GATGAGTCGATCGGACAAGC
EYFPc-V1	forward	GGCTGCAGGTCGACGATGGACAAGCAGAAGAACGG
	reverse	AAACGGCGTCGTGTTCTTGTACAGCTCGTCCATGC
EYFPc-V2	forward	GGAGGTGGTGGAGGCGACAAGCAGAAGAACGGCAT
	reverse	TATCTTGGGTCTAAACTTGTACAGCTCGTCCATGC
EYFPc-V3	forward	TCCGATCGACTCATCGACAAGCAGAAGAACGGCAT
	reverse	CTTAGAATTCCCGGGTTACTTGTACAGCTCGTCCAT
SCM-V1	forward	AGCTTTACAAGATGGGAAGTGTTC
	reverse	CTTAGAATTCCCGGGTTAGATCATATGTTGAAGATCTTGGA
SCM-V2	forward	GGCTGCAGGTCGACGATGAGCTTTACAAGATGGGAAG
	reverse	GATCATATGTTGAAGATCTTGGACT
EYFPn-V1	forward	GGCTGCAGGTCGACGATGGTGAGCAAGGGCGAG
	reverse	CCATCTTGTAAAGCTGGCCATGATATAGACGTTGTG
EYFPn-V2	forward	CTTCAACATATGATCGTGAGCAAGGGCGAGGAG
	reverse	CTTAGAATTCCCGGGTTAGGCCATGATATAGACGTTGTG
