# Supplementary material for: QUIRKY regulates root epidermal cell patterning through stabilizing SCRAMBLED to control CAPRICE movement in Arabidopsis
Source: Nat Commun. 2019 Apr 15;10:1744. doi: 10.1038/s41467-019-09715-8 (PMC6465271; doi:10.1038/s41467-019-09715-8)
Supplement: Supplementary file 3 — Reporting Summary [file 41467_2019_9715_MOESM3_ESM.pdf]

nature research

Corresponding author(s): Myeong Min Lee

Reporting Summary

Nature Research wishes to improve the reproducibility of the work that we publish. This form provides structure for consistency and transparency in reporting. For further information on Nature Research policies, see Authors & Referees and the Editorial Policy Checklist.

Statistical parameters

When statistical analyses are reported, confirm that the following items are present in the relevant location (e.g. figure legend, table legend, main text, or Methods section).

n/a	Confirmed	
☐	☒	The exact sample size (*n*) for each experimental group/condition, given as a discrete number and unit of measurement
☐	☒	An indication of whether measurements were taken from distinct samples or whether the same sample was measured repeatedly
☐	☒	The statistical test(s) used AND whether they are one- or two-sided<br>*Only common tests should be described solely by name; describe more complex techniques in the Methods section.*
☒	☐	A description of all covariates tested
☐	☒	A description of any assumptions or corrections, such as tests of normality and adjustment for multiple comparisons
☐	☒	A full description of the statistics including central tendency (e.g. means) or other basic estimates (e.g. regression coefficient) AND variation (e.g. standard deviation) or associated estimates of uncertainty (e.g. confidence intervals)
☐	☒	For null hypothesis testing, the test statistic (e.g. *F*, *t*, *r*) with confidence intervals, effect sizes, degrees of freedom and *P* value noted<br>*Give P values as exact values whenever suitable.*
☒	☐	For Bayesian analysis, information on the choice of priors and Markov chain Monte Carlo settings
☒	☐	For hierarchical and complex designs, identification of the appropriate level for tests and full reporting of outcomes
☒	☐	Estimates of effect sizes (e.g. Cohen's *d*, Pearson's *r*), indicating how they were calculated
☐	☒	Clearly defined error bars<br>*State explicitly what error bars represent (e.g. SD, SE, CI)*

Our web collection on statistics for biologists may be useful.

Software and code

Policy information about availability of computer code

Data collection	We used IM50, Axiovision Rel. 4.8 and Zen software to collect images on the microscope.
Data analysis	We used Zen software for data analysis, Microsoft Excel for statistical analysis.

For manuscripts utilizing custom algorithms or software that are central to the research but not yet described in published literature, software must be made available to editors/reviewers upon request. We strongly encourage code deposition in a community repository (e.g. GitHub). See the Nature Research guidelines for submitting code & software for further information.

Data

Policy information about availability of data

All manuscripts must include a data availability statement. This statement should provide the following information, where applicable:

- Accession codes, unique identifiers, or web links for publicly available datasets
- A list of figures that have associated raw data
- A description of any restrictions on data availability

The source data for figure 3, figure 4, figure 5, figure 6, figure 7, supplementary figure 4, supplementary figure 5, supplementary figure 6, supplementary figure 8, and table 1 are provided as a Source Data file. All other data that support the findings of this study are available from the corresponding author upon reasonable request.

Field-specific reporting

Please select the best fit for your research. If you are not sure, read the appropriate sections before making your selection.

☒ Life sciences ☐ Behavioural & social sciences ☐ Ecological, evolutionary & environmental sciences

For a reference copy of the document with all sections, see nature.com/authors/policies/ReportingSummary-flat.pdf

Life sciences study design

All studies must disclose on these points even when the disclosure is negative.

Sample size	Table 1: Three independent experiments were carried out. In each experiment, 10 seedlings were examined for each sample except for the qky-16 mutant (eleven 4-day old seedlings were examined for this strain).<br>Figure 3b: Ten seedlings for each line were used and, at least, 20 epidermal cells showing fluorescence were examined.<br>Figure 3c: 70~171 cells were analyzed for CPC movement in each sample.<br>Figure 4f: Protoplasts were isolated from ca. 200~300 seedlings for each sample.<br>Figure 6a,c: Five seedlings were used for each sample, and 10 cells were analyzed in each seedling.<br>Figure 6d: Ten seedlings were used for each sample, and 10 cells were analyzed in each seedling.<br>Figure 6b: Five seedlings were used.<br>Figure 7a,b,e: Ca. 500 seedlings were used for IP experiments.<br>Figure 7d: Three independent experiments were carried out. In each experiment, 5 seedlings were examined for each sample.<br>Supplementary Figure 1f: At least 34 leaves for each sample were analyzed.<br>Supplementary Figure 5c and Supplementary Figure 6a: Total RNA was extracted from the root tips of ca. 500 seedlings for each sample. Each experiment was repeated 3 times, and each time the experiment included triplicate samples.
Data exclusions	No data were excluded.
Replication	Each experiment was reproduced at three times, none were found to be not reproducible.
Randomization	Plants were selected randomly for imaging and protein works.
Blinding	Experiments were not blind because some of the results are quantitative, or not subjective. Blinding was not feasible in most of the experiments.

Reporting for specific materials, systems and methods

Materials & experimental systems

n/a	Involved in the study
☐	☒ Unique biological materials
☐	☒ Antibodies
☒	☐ Eukaryotic cell lines
☒	☐ Palaeontology
☐	☒ Animals and other organisms
☒	☐ Human research participants

Methods

n/a	Involved in the study
☒	☐ ChIP-seq
☒	☐ Flow cytometry
☒	☐ MRI-based neuroimaging

Unique biological materials

Policy information about availability of materials

Obtaining unique materials	All unique materials are available from the authors upon request.

Antibodies

Antibodies used	(Name, Brand, Cat No., Clone, Lot No.)<br>monoclonal anti-GFP, Takara Bio Clonetech, 632380, JL-8, A5033481<br>polyclonal anti-GFP, abcam, ab290, , GR196475-3<br>anti-FK1, Enzolifesciences, BML-PW8805, FK1, 04011678<br>anti-cMYC, Calbiochem, OP10L, 9E10, D00058540

	anti-FLAG, Abm, G191, 1E6, ET7855<br>anti-P4D1, Abcam, ab139101, P4D1, GR261606-10
Validation	N/A

Animals and other organisms

Policy information about studies involving animals; ARRIVE guidelines recommended for reporting animal research

Laboratory animals	N/A
Wild animals	N/A
Field-collected samples	N/A
